# Inhibition of the Citrus Canker Pathogen Using a Photosensitizer Assisted by Sunlight Irradiation

**DOI:** 10.3389/fmicb.2020.571691

**Published:** 2020-11-17

**Authors:** Libin Jiang, Yurong Liu, Xianyuan Xu, Dan Su, Huasong Zou, Jianyong Liu, Cai Yuan, Mingdong Huang

**Affiliations:** ^1^College of Chemistry, Fuzhou University, Fuzhou, China; ^2^State Key Laboratory of Ecological Pest Control for Fujian and Taiwan Crops, Fujian University Key Laboratory for Plant–Microbe Interaction, College of Plant Protection, Fujian Agriculture and Forestry University, Fuzhou, China; ^3^College of Biological Science and Engineering, Fuzhou University, Fuzhou, China

**Keywords:** citrus canker, photosensitizer, antimicrobial activity, photodegradation, sunlight

## Abstract

Citrus canker, induced by bacterial infection, seriously affects the growth and productivity of citrus around the world and has attracted strong research interest. The current treatment for this disease uses copper salts to inactivate the pathogenic bacteria: *Xanthomonas citri subsp. citri* (*Xcc*) strain. However, copper salts may have a negative impact on the environment or plant. In this work, we identify a chemical compound, 2,6-diiodo-1,3,5,7-tetramethyl-8-(P-benzoic acid)-4,4′-difluoroboradiazaindacene (DIBDP), to inactivate the pathogenic *Xcc* strain (*29-1*). DIBDP is activated by sunlight and generates reactive oxygen species to kill the bacteria. In order to overcome the degradation of DIBDP under sunlight, an adjuvant agent was identified to limit the photodegradation of DIBDP by forming a photosensitizer complex (PSC). This complex demonstrated significant antimicrobial activity to *Xcc 29-1*, which was 64-fold more potent than the copper biocides. The antimicrobial efficacy of PSC on citrus leaves infected by *Xcc 29-1* also was much stronger than copper agent and, at the same time, the PSC was safe to the host exposed to sunlight. Thus, this PSC is a promising antibacterial agent to control citrus canker disease.

## Graphical Abstract

TOC: A photosensitizer complex (PSC) was developed to kill the pathogen for citrus canker, *Xanthomonas citri subsp. citri*, under sunlight irradiation, using reactive oxygen species. The PSC is safe to the host leaves and to environment.

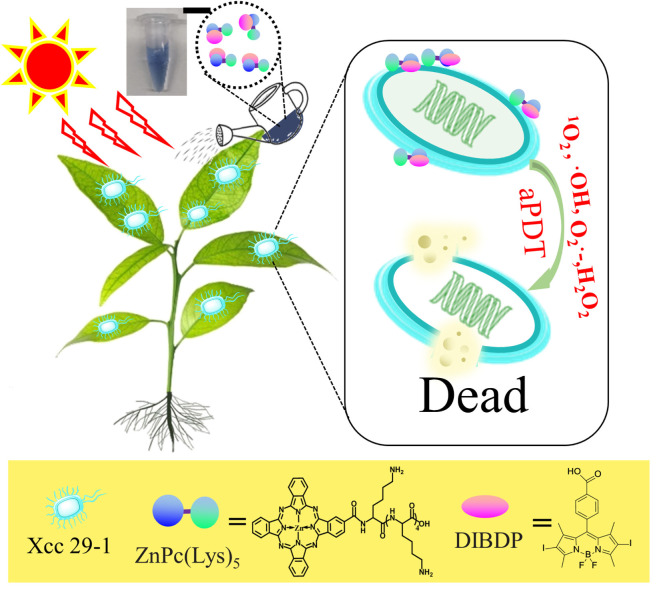

## Introduction

Citrus canker is one of the most serious quarantine diseases worldwide (Gottwald et al., 2002; Brunings and Gabriel, 2003). The canker causes necrotic lesions on leaves, twigs, and fruit, and leads to defoliation and fruit drop in severe cases (Schubert et al., 2001). The canker is caused by the bacterial pathogen *Xanthomonas citri subsp. citri* (*Xcc*), which enters the plant through the stomata or wounds, and invades into the intercellular space in the apoplast (Brunings and Gabriel, 2003; Ryan et al., 2011). The canker spreads out to remote areas, helped by the leaf miner fly or during wind-blown rain (Bock et al., 2005). Although millions of dollars are spent annually on prevention, quarantines, eradication programs, and disease control, citrus canker remains a serious challenge (Graham et al., 2004; Behlau et al., 2016).

Photodynamic therapy is a clinically used method for tumor eradication (Agostinis et al., 2011; Shi et al., 2019). This method has also been used as a new and promising strategy to eradicate a wide spectrum of microorganisms, including bacteria, yeasts, molds, viruses, and parasites (Hamblin and Hasan, 2004; Wainwright et al., 2017), and is named antimicrobial photodynamic therapy (aPDT) (Huang et al., 2010). This therapy requires the presence of a small amount of photosensitizer (PS), typically at a micromolar range concentration. Photosensitizers are often organic dyes, absorb light with long wavelengths (to promote tissue penetration), and transfers light energy to surrounding oxygen, ultimately leading to the generation of reactive oxygen species (ROS), such as singlet oxygen and free radicals (Wainwright, 1998; Huang et al., 2012; Carrera et al., 2016). The ROS causes significant toxicity, leading to death of nearby cells (Sperandio et al., 2013). A strong advantage of aPDT is its very high efficiency to kill the microorganisms, leading to very low chance of them developing resistance (Pedigo et al., 2009; Giuliani et al., 2010; Maisch, 2015).

The translation application of clinical PDT to agricultural PDT to inactivate the citrus canker pathogen has proven to be a major challenge. In clinical PDT, long wavelength light (>630 nm) is used to reach deep tissue (up to ∼10 mm). Photosensitizers satisfying this requirement usually have aromatic rings with a high degree of π electron dislocation in their molecular structure, e.g., porphyrin and phthalocyanine. Such photosensitizers may not be optimal for agriculture PDT, where the light source is sunlight with maximal irradiation at 520 nm (Kim et al., 2015). Thus, a new type of photosensitizer must be found for agricultural PDT.

**FIGURE 1:**
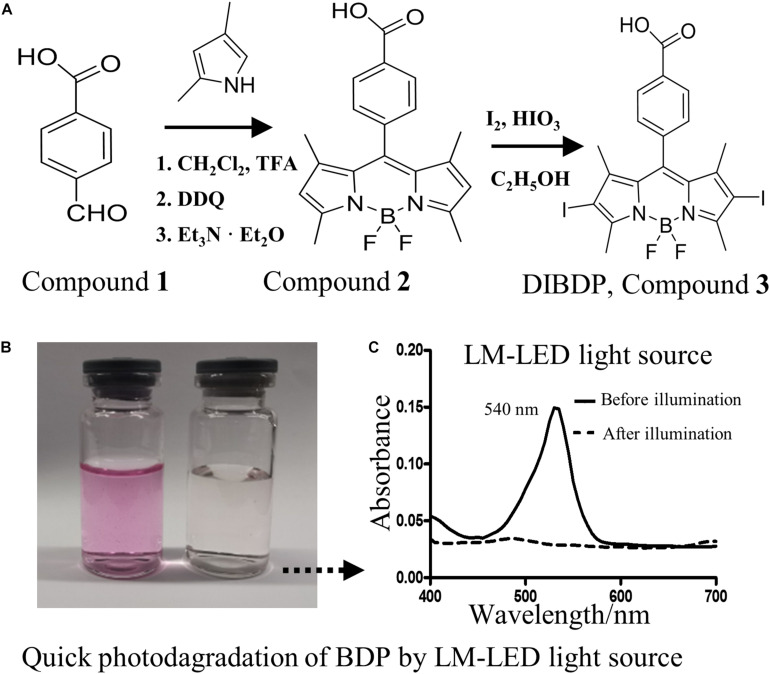
**(A)** Synthesis scheme for the target compound DIBDP. **(B)** Color change before (left) and after (right) light illumination. **(C)** UV-VIS absorption spectrum of DIBDP at 10 μM in PBS before and after light illumination at a dose of 0.25 J/cm^2^ (520 nm, 4 mW/cm^2^).

Organic dye Boron Dipyrromethene (BODIPY), 4,4-difluoro-4-bora-3a,4a-diaza-s-indacene, was discovered in 1968 by Treibs and Kreuzer (Kreuzer, 1968), and consists of two pyrrole units linked by a methine bridge and a BF2 group that connect both pyrrolic nitrogen atoms (Figure 1A). BODIPY derivatives possess remarkable properties, such as high extinction coefficients and high fluorescence quantum yields, and are widely used in many fields, such as in fluorescent markers for bio-imaging and potential photosensitizers in PDT (Loudet and Burgess, 2007; Durantini et al., 2018). Most importantly, BODIPY has an absorption spectrum maximal absorption around 540 nm, close to the Sun’s maximal irradiation at sea level (∼520 nm), and thus can be a candidate for agricultural PDT.

Here, we report an effective and facile method based on aPDT to control citrus canker. We develop a BODIPY-based antibacterial agent that is quite stable under sunlight and highly effective toward *Xanthomonas citri subsp. citri*. This antibacterial agent is about 64 times more effective than the copper agent, representing a new alternative pesticide that controls plant diseases caused by the citrus canker pathogen.

## Materials and Methods

### Materials and Instruments

#### Reagents and Materials

All chemical reagents used were of analytical grade and purchased from Sigma-Aldrich Co., Ltd. (St. Louis, United States) or Sinopharm Chemical Reagent Co., Ltd (Shanghai, China). Pentalysine β-carboxyl phthalocyanine zinc (ZnPc(Lys)_5_), tetra-carboxyl phthalocyanine zinc (Pc(COOH)_4_), and β-carbonyl phthalocyanine zinc (CPZ) were synthesized as described in previous studies (Chen et al., 2006; Dumoulin et al., 2010; Li et al., 2012). Chromatographic purification was performed on silica gel (Qingdao Ocean, Qingdao, Shandong, China, 200–300 mesh) columns with the indicated eluents.

#### Light Source and Instruments

Two different types of light sources were used in the current study. One light source was a LM-LED light (Bridgelux led, Mid Atlantic, United States) which emitted light ranging from 480 to 580 nm with a predominant central wavelength of 520 nm. Light emission spectra of the LM-LED grow light was measured using a FLS 980 fluorescence spectrometer (Edinburgh Instruments, United Kingdom) (Supplementary Figure 1). The other light source was a solar simulator (Newport 91160) with flux approximating natural sunlight irradiance from 295 to 2500 nm.

^1^H-NMR spectra were recorded on an AVANE III 400 (1H, 400 MHz) instrument (Bruker, Karlsruhe, Germany) in CDCl_3_. Chemical shifts were expressed in ppm relative to TMS (0 ppm). Electronic absorption spectra and fluorescence spectra were obtained using a microplate reader (SpectraMax i3x, Molecular Devices Corporation, California, United States).

#### Bacteria Strain and Plants

The *Xanthomonas citri subsp. citri strains 29-1* (*Xcc 29-1*) was cultivated in nutrient broth medium (NB) or nutrient broth supplemented with 1.5% agar (NA) at 28°C, as described in our previous publication (Zou et al., 2011). Honey murcott plants were grown in small pots with sterile soil. Antimicrobial studies were performed in a quarantine greenhouse facility (Fujian University Key Laboratory for Plant–Microbe Interaction, Fuzhou, China) under controlled temperatures (28–35°C) and a relative humidity of 80%.

### Experimental Procedure

#### Synthesis of DIBDP, Compound 3

2,6-Diiodo-1,3,5,7-tetramethyl-8-(P-benzoic acid)-4,4′-difluoro- boradiazaindacene (DIBDP, Compound 3) was prepared using a method similar to ones previously reported (Zhang et al., 2008; Guo et al., 2013). All the reactions were carried out under the atmosphere of nitrogen. The compound 4-carboxylbenzaldehyde (Compound 1, 0.52 g, 3.47 mmol) and 2,4-dimethylpyrrole (0.63 g, 6.63 mmol) were added to anhydrous dichloromethane (500 ml) together with two drops of trifluoroacetic acid. The mixture was stirred overnight at an ambient temperature and was followed by the addition of 2,3-dichloro-5,6-dicyano-p-benzoquinone (DDQ, 0.62 g, 2.74 mmol) in anhydrous dichloromethane and further stirred continuously for 4 h. Under an ice-water bath, triethylamine (18 ml, 0.13 mole) and BF_3_⋅Et_2_O (18 ml, 0.15 mole) were added dropwise into the mixture, and stirred overnight at an ambient temperature. The reaction was monitored by thin-layer chromatography (TLC). After the completion of the reaction, the mixture was washed with saturated NaHCO_3_ aqueous solution, followed by water. The organic fraction was dried over anhydrous Na_2_SO_4_ and then concentrated to dryness under vacuum. The crude product was purified by silica gel column chromatography using CH_2_Cl_2_/petroleum ether (1:2, v/v) as the eluent to make Compound 2 an orange-yellow solid (0.40 g, 39%). Next, I_2_ (0.31 g, 1.21 mmol) and HIO_4_ (0.17 g, 0.98 mmol) were added to a mixture of Compound 2 (0.19 g, 0.53 mmol) in absolute ethanol (200 ml), and then stirred under an atmosphere of nitrogen for 6 h at 60°C. The mixture was concentrated under reduced pressure after the reaction was completed, as monitored by thin-layer chromatography (TLC). Then, the residue was purified by silica gel column chromatography using CH_2_Cl_2_/petroleum ether (1:3, v/v) as the eluent to give DIBDP as a red solid (0.25 g, 85%), and confirmed by ^1^H NMR (400 MHz, CDCl_3_).

#### Photostability Measurement of DIBDP

The DIBDP (10 μM) in PBS was illuminated using the LM-LED light source at a light dosage of 4 mW/cm^2^ or the solar simulator at a power density of 80 mW/cm^2^. The ultraviolet-visible spectrum of DIBDP was monitored at 540 nm on a microplate reader.

#### Preparation of Stable Photosensitizer Complex (PSC) Under Light Irradiation

To prepare a stable photosensitizer complex under light irradiation, three adjuvants (1 mM in DMSO) – pentalysine β-carboxyl phthalocyanine zinc (ZnPc(Lys)_5_), tetra-carboxyl phthalocyanine zinc (Pc(COOH)_4_), and β-carbonyl phthalocyanine zinc (CPZ), were respectively mixed with DIBDP (1 mM in DMSO) at a molar ratio of 1:1. The mixed solutions were added into PBS buffer up to 1 ml, followed by stirring for 2 h. The DMSO was then removed by dialysis against proper solvents (PBS or DI water) overnight at room temperature. The stability of the samples was evaluated using the ultraviolet-visible spectrum of DIBDP with illumination.

A similar procedure was employed to optimize the amount of adjuvant ZnPc(Lys)_5_ needed. DIBDP was mixed with the adjuvant at different molar ratios (10:1, 5:1, 2:1, 1:1, 1:2, 1:5, and 1:10). The absorbance value of DIBDP at 540 nm was monitored during constant illumination for 720 s. The photodegradation rate of DIBDP was assessed using the photodegradation rate constant K, following the formula: $N_{t}=N_{0}^{*}\,{\text{e}}^{-\mathrm{kt}}$ (Eggeling et al., 1999; Demchenko, 2020). Here, N_*t*_ and N_0_ was residual DIBDP of the experimental and control group, respectively, and K was the photodegradation rate constant. This is a popular kinetic approach used to quantify photobleaching, based on the assumption that photobleaching is a quasiunimolecular reaction, and the concentration of the dye molecule shows an exponential decrease in time from the initial concentration.

#### Antimicrobial Studies of PSC on *Xcc 29-1*

*Xcc 29-1* was cultivated in nutrient broth medium at 28°C until reaching10^8^ CFU/ml, and then diluted to ∼10^6^ CFU/ml in PBS. The diluted *Xcc 29-1* suspension was added into 96-well plates with 200 μl per well and incubated with the PSC solution at different concentrations (10^−4.5^ M, 10^−5^ M, 10^−5.5^ M, 10^−6^ M, 10^−6.5^ M, 10^−7^ M, 10^−7.5^ M, 10^−8^ M, 10^−8.5^ M, 10^−9^ M and 10^−9.5^ M, respectively). Four replicates at each concentration were tested. The plate was illuminated using the solar simulator to a power density of 80 mW/cm^2^ (1 min). The number of alive bacteria was evaluated by colony counting method. Bacterial solution (100 μl) from each well was serially diluted to 10^–1^ to 10^–5^ in PBS and spread onto nutrient broth agar plates. After incubation at 28°C for 48 h, the colonies were counted and the survival percentage was calculated as the average number of colonies of the treated plates divided by the average number of colonies of the control plate.

#### Determination of MIC Against *Xcc 29-1*

The antibacterial potential of PSC was compared to copper sulfate and copper hydroxide. Minimum inhibitory concentration (MIC) values were measured using the double dilution method according to our previously reported work or others with some modifications (Liu et al., 2018; Rodrigues et al., 2018). *Xcc 29-1* was grown in NB medium at 28°C with constant shaking at 200 rpm to an O.D 600 of 0.3 and was adjusted to a concentration of 10^6^ colony-forming units ([CFU]/ml). 100 μl of such nutrient broth was pipetted into a set of wells in a 96-well microplate. In another set of wells, 100 μl of a 248 μg/ml stock of PSC was added and serially diluted to concentrations of 124, 62, 31, 15.5, 7.75, 3.9, 1.9, 0.97, 0.48, 0.24, and 0.12 μg/ml. Copper sulfate or copper hydroxide concentration gradient (2,000, 1,000, 500, 250, 125, 62.5, 31.25, 15.6, 7.8, 3.9, and 1.96 μg/ml) were also set up. The first column of wells containing only broth was used as negative control. A 100 μl aliquot of 5 × 10^6^ CFU/ml bacteria suspension was added to each well. Then, the PSC was illuminated by the solar simulator to a light dosage of 4.8 J/cm^2^. The microplate was incubated in 28°C at 200 rpm. After 24 h, the MIC concentration was established as the lowest concentration of the compound in which *Xcc 29-1* did not grow. All determinations were conducted in three replicates and repeated three times.

#### Antibacterial Mechanism of PSC

##### Morphologies change of Xcc 29-1 treated by PSC

The morphologies of *Xcc 29-1* treated by PSC were observed using a scanning electron microscope (SEM). The specimens of *Xcc 29-1* were prepared by the procedure of fixation, dehydration, and coating. For details, *Xcc 29-1* was harvested by being centrifuged at 6,000 *g* for 10 min and washed twice with sterile phosphate buffered saline (PBS). For fixation, the bacteria were fixed with pre-cooling 2.5% (v/v) glutaraldehyde in PBS overnight at 4°C, then washed by PBS twice. For dehydration, the bacteria were soaked sequentially in a series of ethanol (30, 50, 70, 90, and 100%) for about 10–15 min at each concentration. For the coating and observation, the dehydrated bacteria were placed onto silicon wafers and dried at 37°C overnight. Then, prepared specimens were sprayed with gold before observation on a scanning electron microscope (SEM).

##### ROS measurement of PSC

Detection of ROS was performed using the probe 2,7-dichlorofluorescein diacetate (DCFH-DA), which can be transformed into 2,7-dichlorofluorescein (DCF, ex 488 nm, em 525 nm) in the present ROS. Ascorbic acid is a well-known water-soluble antioxidant and is a chemically scavenged singlet oxygen (Chou and Khan, 1983). In the present study, PSC (5 μM) and DCFH-DA (100 μM) were added to 96-well plates with or without ascorbic acid (100 μM), giving total volumes of 200 μl. The solutions were irradiated using the solar simulator at a power density of 80 mW/cm^2^ for 8 min. DCF fluorescence intensity (excited at 488 nm) was monitored on a microplate reader (PerkinElmer Instruments).

#### Stability Studies of the PSC on Leaves

Plant leaves of approximately the same size were placed in a 12-well plate, and 20 μl of PSC stock solution (50 μM) was pipetted onto the leaves, forming liquid droplets. The leaves were illuminated using the solar simulator at a power density of 80 mW/cm^2^ for different amounts of time. The PSC solutions on leaves were then recovered. The leaves were further washed with DMSO solution. The DIBDP in the combined solutions was quantified by measuring the absorption at 540 nm. We have carried out a control experiment to show that DMSO extraction on plain leaves did not display 540 nm absorption.

#### Antimicrobial Studies of the PSC on Leaves

##### Puncture inoculation

In order to determine the curative activities of PSC in citrus plants, well growing citrus leaves were chosen and inoculated using the puncture method. Fully extended leaves (*n* = 15) were randomly divided into three groups and 10 pin-holes were punctured per leaf. *Xcc 29-1* cells at a final concentration of 10^8^ CFU/ml were infiltrated into citrus leaves with degreasing cotton. A total of 1 day after inoculation, the PSC solution at 30 μg/ml (50 μM) and copper sulfate solution at 1 mg/ml were uniformly sprayed onto the leaves until dripping down, whereas PBS solution was uniformly sprayed onto the negative control leaves. The leaves of the PSC group were illuminated by the solar simulator with a power density of 80 mW/cm^2^ for 10 min. At 7 and 14 days after spraying, using a macroscopic lesion for observation, the disease development was recorded and disease incidence was calculated. The disease incidence of puncture inoculation was calculated by dividing the total infected leaves with total inoculated.

##### Spray infection

In these preventive assays, the leaves of sweet orange were kept under greenhouse conditions and were sprayed with either the PSC solution at 30 μg/ml or copper sulfate solution at 1 mg/ml. Upon drying of the leaf surface (∼2 h), the Xcc 29-1 culture suspensions (10^8^ CFU/ml) were sprayed on leaves (18 leaves per strain and randomly divided into three groups) until fully covered with bacterial suspension. A PBS treatment was setup as a negative control. Each group had three replicates. The PSC treatment group was illuminated by the solar simulator at a power density of 80 mW/cm^2^ for 10 min. At 30 days post-inoculation, the disease severity of each group was measured regarding the number of citrus canker lesions per cm^2^, and foliar area were measured using digital images from Adobe Photoshop software (Adobe Systems Inc., San Jose, CA, United States).

In the curative assays, the PSC solution (30 μg/ml) or copper sulfate solution (1 mg/ml) at 10 μl/lesion were added to 35-day-old canker lesions of leaves, which generated in the preventive tests. The PSC treatment group was illuminated by the solar simulator with a power density of 80 mW/cm^2^ for 10 min. After a day, the treated leaves were disinfested by immersion in 70% ethanol for 1 min, followed by washing with sterilized distilled water for 1 min. Each individual lesion (with a size ∼4 mm^2^) was cut out and smashed in 1 ml PBS with a sterile glass rod. The bacterial suspension (100 μl) was serially diluted to 10^–1^ to 10^–5^ in PBS and spread onto nutrient broth agar plates. After incubation at 28°C for 48 h, the number of cfu were counted and transformed to log10 cfu/lesion.

## Results

### Design and Characteristic of DIBDP

We chose a DIBDP photosensitizer (Figure 1A) to study its ability to inactivate bacteria based on the following considerations. First, this photosensitizer has a strong photodynamic effect due to the presence of iodine atoms. The heavy atoms (iodine) facilitate intersystem crossing of excited photons, therefore promoting the generation of singlet oxygen (a type of ROS) in high efficiency. Secondly, the compound has a maximal adsorption at 540 nm in its UV-VIS absorption spectrum (Figure 1B), which matches the maximal emission wavelength of sunlight (Kim et al., 2015). This DIBDP compound was synthesized chemically in high yield (Figure 1A), and its structure was fully confirmed by proton NMR spectrum (Supplementary Figure 2).

However, we found that the DIBDP degraded rapidly under light illumination. The DIBDP at a concentration of 10 μM completely degraded in 1 min with the illumination of an LED light source (4 mW/cm^2^) or a solar simulator (80 mW/cm^2^) (Figure 1C and Supplementary Figure 3).

### Optimization of a Photosensitizer Complex (PSC) Stable Under Sunlight

**FIGURE 2:**
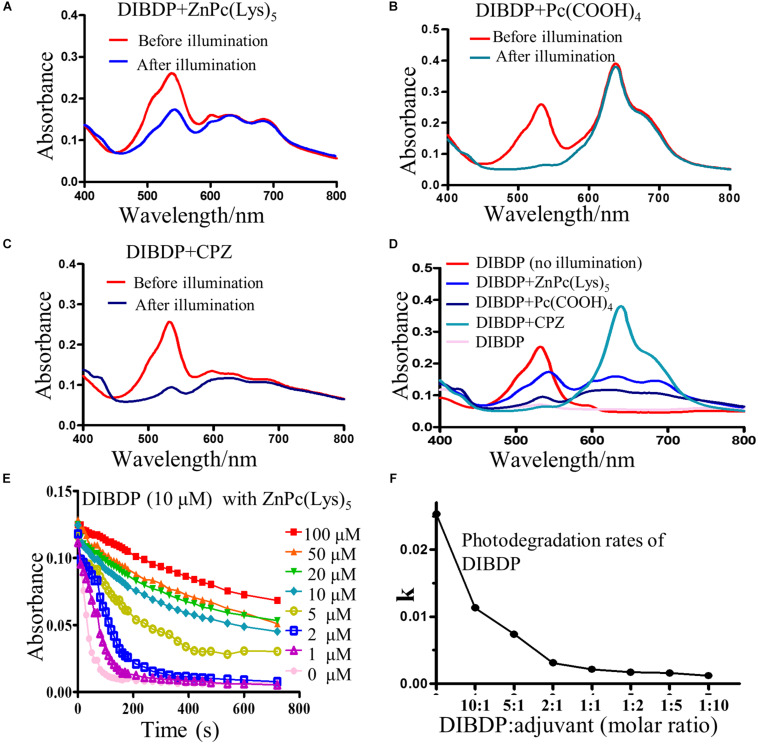
Optimization of a complex (PSC) for resistance to photodegradation under sunlight irradiation. **(A–C)**: The absorbance spectrum of DIBDP with three adjuvants at 1:1 molar ratio in PBS buffer before or after light irradiation (520 nm, 1 min at 4 mW/cm^2^); [**(A)**, pentalysine β-carboxyl phthalocyanine zinc (ZnPc(Lys)_5_]; **(B)** tetra-carboxyl phthalocyanine zinc (Pc(COOH)_4_); [**(C)** β-carbonyl phthalocyanine zinc (CPZ)]. **(D)** Comparison of the stabilizing effects of three adjuvants. **(E)** Typical photodegradation kinetics to optimize molar ratio of DIBDP (10 μM) and the adjuvant (ZnPc(Lys)_5_) (1 μM to 100 μM) by monitoring absorbance value of DIBDP at 540 nm for 720 s with a LM-LED light source (4 mW/cm^2^). **(F)** The quantification of **(E)** into photodegradation rate constant (k).

To meet the challenge of photodegradation, we searched for an adjuvant to increase the DIBDP photostability under sunlight. In our previous work, we found that a compound named zinc phthalocyanine is quite resistant to photodegradation, despite being an organic dye (Jia et al., 2018). In this work, we studied a series of this type of compound to see if they can stabilize the DIBDP we chose. We selected a number of zinc phthalocyanine compounds, mixed them with the DIBDP, and measured the photodegradation rates of the mixtures at different molar ratios. We chose three compounds with different characteristics: β-carbonylphthalocyanine zinc (CPZ), tetra-carboxyphthalocyanine zinc (Pc(COOH)_4_), and pentalysine β-carboxyl phthalocyanine zinc (ZnPc(Lys)_5_). Fortunately, we did identify a compound (ZnPc(Lys)_5_) that stabilized the photobleaching of DIBDP (Figures 2A–D).

In order to optimize the amount of the adjuvant needed, we mixed the DIBDP (10 μM) with different amounts of the adjuvant (1 μM to 100 μM) and measured the photodegradation rate. Figure 2E showed the effect of the adjuvant on the DIBDP photobleaching rates. Illumination of DIBDP in solutions leads to quick photobleaching: more than 90% degradation in 100 s and almost complete degradation in 200 s for 10 μM of DIBDP at a light illumination condition of 4 mW/cm^2^. The degradation of DIBDP reduced with the increase of adjuvant concentration. Even a small amount of adjuvant (1 μM) reduced the photodegradation rate of DIBDP by 50% (Figure 2E), demonstrating the power of this method. At the 1:1 molar ratio, the photodegradation rate reduced nearly 12-fold. In addition, PSC showed a similar result under the condition of sunlight (Supplementary Figures 4A,B). In the following experiments, we selected a molar ratio of DIBDP:adjuvant of 1:2, which led to the optimal 15-fold reduction of DIBDP photodegradation (Figure 2F). This agent is here named photosensitizer complex or PSC. We want to point out that the adjuvant agent, zinc phthalocyanine, has absorption at the far red region (Kobayashi et al., 2003), and thus does not interfere with the photodynamic effect of the DIBPD. The minimum inhibitory concentration (MIC) of zinc salt or its metal against bacteria was very high (500 μg/ml, or 3.12 mM) (Goncalves et al., 2017; Gugala et al., 2019), much higher than the concentration of adjuvant that we used here. Thus, it is unlikely that the antimicrobial efficacy of PSC observed here was due to the zinc salt in the adjuvant. Stability of PSC on leaves upon light illumination.

We also evaluated the stability of PSC on citrus leaves using the solar simulator (Supplementary Figure 5). The DIBDP itself on leaves again led to quick photobleaching and almost complete degradation in 6 min. On the other hand, PSC showed excellent resistance with over 60% of intact BDP left at 40 min. With longer light illumination, the PSC solution evaporated completely and become a solid film after 25 min, and the PSC solid was no longer degraded.

### Antibacterial Efficacy of PSC Under Sunlight

**FIGURE 3:**
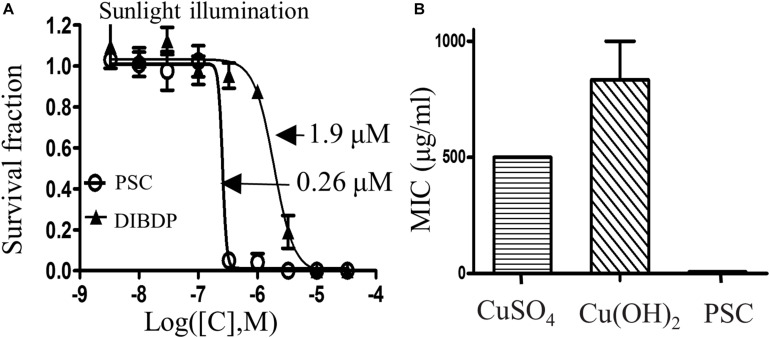
Antimicrobial effects of PSC on *Xcc 29-1*. **(A)** The photoinactivation of DIBDP or PSC against *Xcc 29-1* under sunlight irradiation with the solar simulator at a power density of 80 mW/cm^2^ (1 min). **(B)** Minimum inhibitory concentration (MIC) of PSC, CuSO_4_, and Cu(OH)_2_ against *Xcc 29-1*.

The antibacterial effect of the PSC against the *Xcc 29-1* bacteria strain was measured using the colony counting method with light illumination from a solar simulator at the power of 80 mW/cm^2^. The IC_50_ of the PSC was measured to be 0.26 μM, which was 7.3-fold lower than that of DIBDP alone (1.9 μM), showing enhanced antimicrobial activity in the presence of the adjuvant agent (Figure 3A).

Copper salts are currently used to control the infection by Xcc, even though their potencies are low. We measured the minimum inhibitory concentration (MIC) of the PSC toward the *Xcc 29-1* strain, together with the positive control copper sulfate and copper hydroxide, two widely used agents to control citrus canker. The PSC showed a 64-fold lower MIC against *Xcc 29-1* (MIC 7.75 μg/ml) compared to copper sulfate (MIC 500 μg/ml) or copper hydroxide (MIC 830 μg/ml) (Figure 3B). These results demonstrated that the PSC was much more potent against *Xcc 29-1* than the copper salts.

### Antibacterial Mechanism of PSC

**FIGURE 4:**
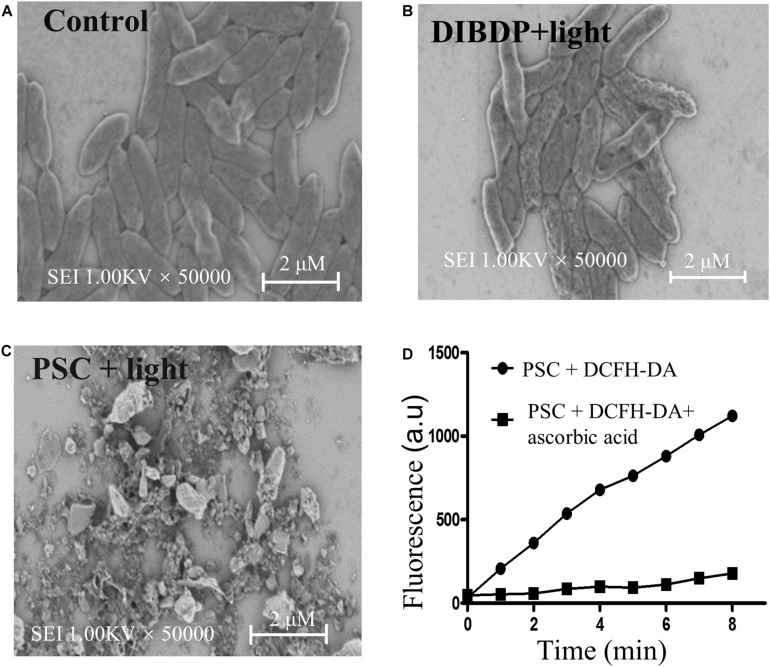
Antibacterial mechanism of PSC. SEM images of *Xcc 29-1* before **(A)** and after treatment with DIBDP **(B)** or PSC **(C)** at 10 μM followed by sunlight irradiation. **(D)** Fluorescence of DCFH-DA (100 μM) was activated by ROS generated by PSC (5 μM), but quenched by ascorbic acid (100 μM).

To further study how the PSC damage bacteria cell’s surface morphology, scanning electron microscopy (SEM) was performed on *Xcc 29-1* after incubation with PSC with illumination. As shown in Figure 4A, *Xcc 29-1* without treatment displayed characteristic straight rods with smooth outer surfaces. In contrast, the morphology of the bacteria cell showed membrane deformation and surface collapse after treatment with DIBDP at a concentration of 10 μM and light irradiation (Figure 4B). With treatment of PSC, the bacteria practically broke down into debris, as shown by SEM (Figure 4C). These results confirmed the severe damage of *Xcc 29-1* induced by aPDT under light irradiation, and the PSC enhanced antimicrobial activity compared to free DIBDP.

Next, we measured the generation of ROS from the PSC using the probe DCFH-DA, which has no fluorescence but is converted to fluorescent species (DCF) in the presence of ROS. As shown in Figure 4D, the DCF fluorescence intensity was gradually increased as the irradiation time was prolonged. Such fluorescence was suppressed in the presence of ascorbic acid, which is a relatively specific quencher for singlet oxygen (^1^O_2_). These experiments demonstrated that PSC inactivated the pathogen using ROS, most likely by singlet oxygen.

### Effect of PSC on Citrus Canker Development

In order to evaluate the antimicrobial activity of the PSC on plants infected with citrus canker, both puncture inoculation and spray infection methods were carried out, using 30 μg/ml PSC for treatment, based on the MIC value measured above. Copper sulfate (1 mg/ml) was used as control.

**FIGURE 5:**
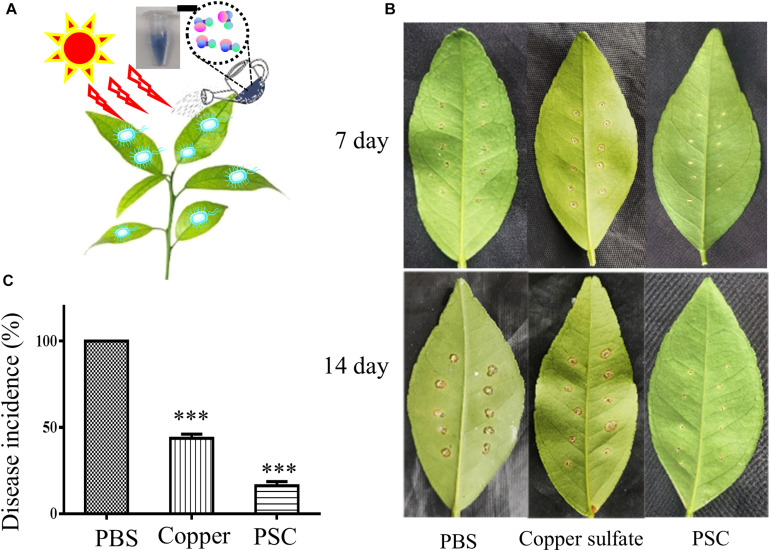
Antimicrobial effect of PSC on citrus plants infected with *Xcc* bacteria. **(A)** A photosensitizer complex (PSC) was developed to kill the pathogen for citrus canker, *Xanthomonas citri subsp. citri*, under sunlight irradiation, using reactive oxygen species. **(B)** Representative Honey murcott leaves treated with PBS, copper sulfate, or PSC at 7 and 14 day using the puncture inoculation method. **(C)** Quantitative results of three treated groups. All bars represent standard error of the mean (SEM). The data were analyzed for statistical differences using one-way ANOVA (^∗∗∗^*P* < 0.001).

A puncture inoculation plant model was established by inoculating *Xcc 29-1* bacteria on perforated leaves. Lesion development on the leaves was monitored daily up to 14 days after inoculation. In our assay, a solution of the PSC (30 μg/ml) was uniformly sprayed onto the leaves after the application of *Xcc 29-1* inoculum. The solvent (PBS) and copper sulfate (1 mg/ml) were used as the negative and positive control, respectively (Figure 5A). After the inoculation of *Xcc 29-1* bacteria for 5 days, the leaves began to show spongy pustules. On the 7th day, these pustules darkened and thickened into a light tan to brown corky canker, which was rough to the touch (Figure 5B). These corky cankers were further aggravated and all the punctures developed into severe corky cankers by the 14th day. The PSC treatment remarkably reduced lesion development over time compared with the controls. In addition, the PSC treatment showed results superior to the copper sulfate treatment group. The copper control group had 45% punctures that developed into corky cankers by the 14th day and showed severe damage to the citrus leaves. The PSC treatment significantly reduced the incidence of canker lesions, with only 16% of punctures deteriorating into cankers (Figure 5C).

**FIGURE 6:**
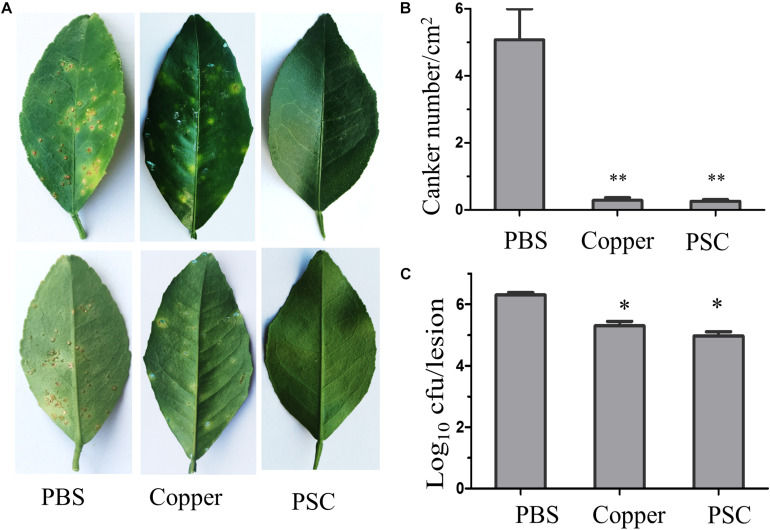
Preventive **(A,B)** and curative **(C)** effects of PSC in citrus canker control. **(A)** The canker lesions developed on leaf surface 30 days after being spread with *Xcc 29-1* (top panel, PBS group) but had much less infection in the group treated with either copper sulfate (1 mg/ml) or PSC (30 μg/ml). The quantitation of canker number was shown in **(B)**. **(C)** The number of CFU in each lesion at 35-day-old canker lesions after treatments with either copper salt or PSC. All bars represent standard error of the mean (SEM). The data were analyzed for statistical differences using one-way ANOVA (^∗^*P* < 0.05, ^∗∗^*P* < 0.01).

We also evaluated the preventive and curative effects of the PSC based on spray infection, which reflected the natural infection process of citrus canker. It took about 30 days post spread inoculation with wild type Xcc 29-1 for the canker lesion to develop. The lesions had a typical brownish corky-like appearance. The number of citrus canker lesions/cm^2^ on leaves treated with PBS was an average of 5.07 lesions/cm^2^, while the PSC treatment group had only an average of 0.26 lesions/cm^2^ on the leaves (Figures 6A,B). These results clearly indicated that the PSC (P < 0.001) strongly prevented canker development and is useful for the control of canker in citrus fruit plantations. In our control, copper salt also showed decent effect in preventing the development of citrus canker lesions (Figure 6). For the curative assay, we applied either PSC or copper to the infected leaves, and measured the amount of bacteria on 15 lesions in each group by CFU counting. The result showed the amount of bacterial population on the leaves of trees treated with PSC was reduced by 95%, similar to the copper treatment, demonstrating that PSC is a promising agent to control citrus canker (Figure 6C).

The above results indicated that PSC killed *Xcc 29-1* in plants under sunlight at a concentration much lower than copper (30 μg/ml vs. 1 mg/ml). In addition, we observed no adverse effects at all of PSC on the normal plant leaves at the doses used to kill *Xcc 29-1* bacteria, demonstrating the safety of PSC.

## Discussion

There is strong interest in understanding the pathogenesis of *Xcc* and its interaction with its host in hope to find new agents to intervene with citrus canker formation (Ference et al., 2018). We identified a number of novel genes of *Xcc* (Song et al., 2015; Xia et al., 2016) that are related to its virulence. One of them encodes an extracellular endoglucanase on *Xcc* (BglC3) and is required for the full virulence of *Xcc* (Xia et al., 2016). In addition, a response regulator (VemR) was found to be important in the flagellum-derived cell motility of *Xcc* (Wu et al., 2019). Deletion of this gene (*vemR*) reduced not only cell motility, but also the exopolysaccharide production, leading to lower virulence. This VemR was also an RpoN2 cognate activator and positively regulated the transcription of the rod gene *flgG* in the bacteria (Wu et al., 2019). These works identified critical genes on *Xcc* for citrus infection that could be valuable targets to control citrus canker. In another strategy to control citrus canker, an *Xcc* resistant strain of citrus was generated by engineering of the *Xcc*-susceptibility gene *CsLOB1* of citrus using CRISPR/Cas9-mediated promoter editing (Peng et al., 2017). The current management methods of citrus canker mainly include the applications of copper agents to kill or inhibit pathogenic bacteria, insecticide to control the leaf miner fly which rapidly spreads canker diseases (Leite and Mohan, 1990), or biological control agents such as bacteriophages (Balogh et al., 2008; Ibrahim et al., 2017). Bacteriophage suffers from having a short active period caused mainly by the detrimental effects of sunlight UV irradiation, seriously affecting its control effect (Ji et al., 2006).

Copper bactericide is the main agent used in large quantities despite its low antibacterial efficacy (Marin et al., 2019). Long-term use of copper bactericides results in a harmful impact on the environment, plants, and safety human (Zhu and Alva, 1993; Lin et al., 2010). In addition, the multiple and independent applications of chemical pesticides have led to the emergence of resistance genes identified in Argentina and Florida, United States (Behlau et al., 2011, 2013). Aggressive measures to remove infected trees can only prevent the diffusion of the pathogen to some extent and cannot completely control citrus canker (Sosnowski et al., 2009). The lack of effective control methods seriously damage the citrus industry and result in significant economic losses. Therefore, it is desirable to develop an effective and ecologically friendly anti-microbial technology to replace copper pesticides.

In the present study, we developed a photosensitizer complex (PSC) to inactivate *Xcc 29-1* under sunlight. The result showed that *Xcc 29-1* was effectively inactivated at a MIC value of 7.75 μg/ml PSC, much lower than the copper sulfate control (500 μg/ml).

Our results of PSC showed low toxicity to leaves when exposed to sunlight. Such safety to leaves could be due to the low concentration (μM range) of PSC used during aPDT. Moreover, the lifetime of single oxygen in tissues is very short (<40 ns) and its action distance is limited (less than 20 nm) (Moan, 1990). In addition, the leaf cuticle of the citrus leaves appears to be more resistant to aPDT using PSC. Such resistance in plants is in major contrast to animal cells and microorganisms, which are more vulnerable to insults from PDT.

The development of bacterial resistance to aPDT was considered unlikely due to the multi-target mechanism of photoinactivation (Tavares et al., 2010; Martins et al., 2018). Thus, the application of aPDT to control citrus canker could be a safe alternative to copper for the effective control of canker in citrus fruit plantations. In the long run, PSC will be degraded over time in the environment, which should reduce the risk of its accumulation. Future studies will include field trials’ evaluation and investigation of its environmental fate on non-target species.

## Conclusion

In conclusion, we successfully prepared a photosensitizer complex (PSC) and demonstrated its antibacterial effects using a solar simulator on citrus leaves. The PSC was prepared by simple mixing; the optimum mixing ratio of DIBDP and adjuvant agent was 1:2. Under sunlight irradiation, PSC had more enhanced antimicrobial activity than single DIBDP. Compared to the traditional antibacterial agent copper salts, PSC showed a lower MIC concentration (MIC 7.75 μg/ml) against *Xcc 29-1* comparable to copper standards (MIC 500 μg/ml). The PSC showed much strong efficacy than the copper agent and had lower toxicity to leaves, demonstrating that PSC is a promising agent to control citrus canker.

## Data Availability Statement

All datasets presented in this study are included in the article/Supplementary Material.

## Author Contributions

LJ carried out the experiments and drafted the manuscript. YL, XX, and DS assisted in the experiments. HZ and JL provided key reagents and expertise necessary for this interdisciplinary work. CY and MH conceived and designed the project, analyzed data, and wrote the manuscript. All authors contributed to the article and approved the submitted version.

## Conflict of Interest

The authors declare that the research was conducted in the absence of any commercial or financial relationships that could be construed as a potential conflict of interest.
