# Supplementary material for: Inhibition of the Citrus Canker Pathogen Using a Photosensitizer Assisted by Sunlight Irradiation
Source: Front Microbiol. 2020 Nov 17;11:571691. doi: 10.3389/fmicb.2020.571691 (PMC7705355; doi:10.3389/fmicb.2020.571691)
Supplement: Supplementary file 1 [file Data_Sheet_1.pdf]

Supplementary Material

Supplementary Figures

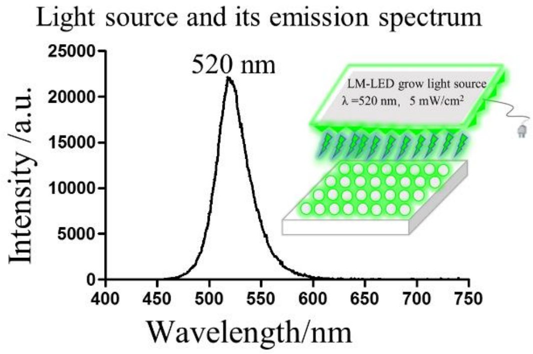

Supplementary Figure 1. Light emission intensity distribution of a LM-LED light source used in present study.

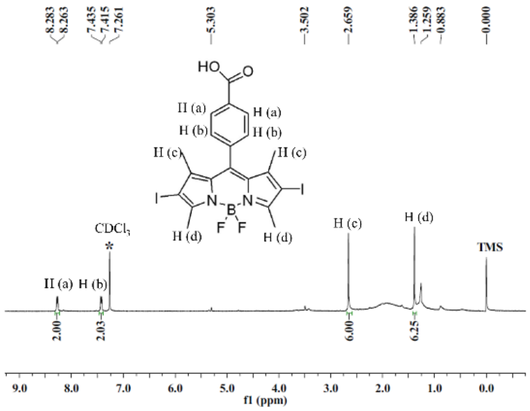

Supplementary Figure 2. ^{1}H NMR spectrum and the assignment of chemical shifts of target DIBDP. ^{1}H-NMR (400 MHz, CDCl3): δ = 8.27 (d, J = 8.0 Hz, 2H, ArH), δ = 7.43 (d, J = 8.0 Hz, 2 H, ArH), δ = 2.66 (s, 6 H, CH_3), 1.39 (s, 6 H, CH_3).

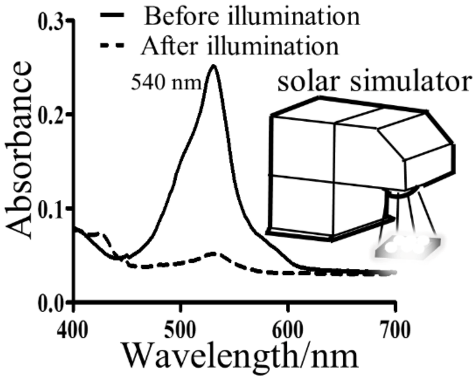

Supplementary Figure 3. UV-VIS absorption spectrum of DIBDP at 10 µM in PBS before and after light irradiation with a solar simulator power at a density of 80 mW/cm^2 for 1 min and the solar simulator light source used in this assay (inset).

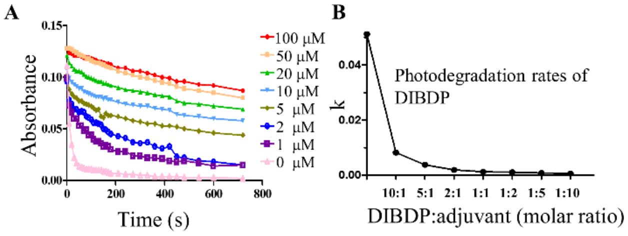

Supplementary Figure 4. (A) Typical photodegradation kinetics was used for optimizing the molar ratio of DIBDP (10 µM) and ZnPc(Lys)$_5$ (1 µM to 100 µM) by monitoring absorbance value of DIBDP at 540 nm for 720 seconds with a solar simulator (80 mW/cm^2). (B) Quantification of (A) in term of photodegradation rate constant (k).

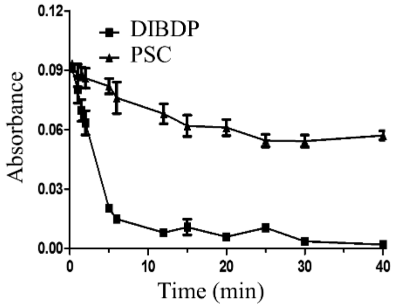

Supplementary Figure 5. Stability of PSC on leaves upon light illumination monitored at

540 nm after illumination with a solar simulator power at a density of 80 mW/cm^2 for amount of different time.
